# The Action of Red Cell Calcium Ions on Human Erythrophagocytosis *in Vitro*

**DOI:** 10.3389/fphys.2017.01008

**Published:** 2017-12-04

**Authors:** Pedro J. Romero, Concepción Hernández-Chinea

**Affiliations:** Laboratory of Membrane Physiology, Faculty of Sciences, Institute of Experimental Biology, Central University of Venezuela, Caracas, Venezuela

**Keywords:** erythrophagocytosis, internal calcium, autologous serum, red blood cells, Gárdos channel

## Abstract

In the present work we have studied *in vitro* the effect of increasing red cell Ca^2+^ ions on human erythrophagocytosis by peripheral monocyte-derived autologous macrophages. In addition, the relative contribution to phagocytosis of phosphatidylserine exposure, autologous IgG binding, complement deposition and Gárdos channel activity was also investigated. Monocytes were obtained after ficoll-hypaque fractionation and induced to transform by adherence to glass coverslips, for 24 h at 37°C in a RPMI medium, containing 10% fetal calf serum. Red blood cells (RBC) were loaded with Ca^2+^ using 10 μM A23187 and 1 mM Ca-EGTA buffers, in the absence of Mg^2+^. Ca^2+^-loaded cells were transferred to above coverslips and incubated for 2 h at 37°C under various experimental conditions, after which phagocytosis was assessed by light microscopy. Confirming earlier findings, phagocytosis depended on internal Ca^2+^. Accordingly; it was linearly raised from about 2–15% by increasing the free Ca^2+^ content of the loading solution from 0.5 to 20 μM, respectively. Such a linear increase was virtually doubled by the presence of 40% autologous serum. At 7 μM Ca^2+^, the phagocytosis degree attained with serum was practically equal to that obtained with either 2 mg/ml affinity-purified IgG or 40% IgG-depleted serum. However, phagocytosis was reduced to levels found with Ca^2+^ alone when IgG-depleted serum was inactivated by heat, implying an involvement of complement. On the other hand, phagocytosis in the absence of serum was markedly reduced by preincubating macrophages with phosphatidylserine-containing liposomes. In contrast, a similar incubation in the presence of serum affected it partially whereas employing liposomes made only of phosphatidylcholine essentially had no effect. Significantly, the Gárdos channel inhibitors clotrimazole (2 μM) and TRAM-34 (100 nM) fully blocked serum-dependent phagocytosis. These findings show that a raised internal Ca^2+^ promotes erythrophagocytosis by independently triggering phosphatidylserine externalization, complement deposition and IgG binding. Serum appeared to stimulate phagocytosis in a way dependent on Gárdos activity. It seems likely that Ca^2+^ promoted IgG-binding to erythrocytes via Gárdos channel activation. This can be an important signal for clearance of senescent human erythrocytes under physiological conditions.

## Introduction

The normal human red blood cell (RBC) ages in the blood stream while circulating ceaselessly for a finite lifespan of nearly 120 days (Berlin and Berk, 1975). Aging appears as a continuous process of multifactorial origin, becoming abruptly interrupted by splenic retention of the senescent RBC. The sequestered cell is immediately recognized and phagocytosed by resident macrophages (Rifkind, 1966; Mebius and Kraal, 2005). Although many different hypotheses have accumulated over the years, the mechanisms responsible for trapping, recognition and destruction of aged cells are not completely elucidated (Clark, 1988; Bratosin et al., 1998; Antonelou et al., 2010; Lutz and Bogdanova, 2013). Suggested age-related changes involved in these mechanisms include dehydration with augmented cell density, decreased size, vesiculation, increased oxidative stress, band 3 clustering, band 3 phosphorylation, increased membrane IgG content and loss of membrane phospholipid asymmetry (Low et al., 1985; Lutz et al., 1987; Ciana et al., 2004; Willekens et al., 2008; Franco et al., 2013; Lutz and Bogdanova, 2013).

One of the current hypotheses that has great consensus is based upon IgG attachment to the RBC membrane and its recognition for macrophage clearance via Fc receptor interaction (Arese et al., 2005; Bosman et al., 2005; Lutz and Bogdanova, 2013). It is well-known that IgG accumulates on the outer membrane surface as the RBC ages (Lutz and Stringaro-Wipf, 1983). A small fraction of IgG binds significantly to α-galactosyl residues of presumably membrane glycolipids in aged RBCs (Galili et al., 1984). Another fraction is bound to a neo-antigenic region located on band 3 protein, which becomes progressively expressed during cell aging. This region corresponds to a distinct interdimeric band 3 epitope exposing binding sites for low-affinity autologous IgG, which is formed upon oligomerization and clustering of band 3 aggregates during RBC senescence (Lutz et al., 1984; Kannan et al., 1991; Lutz, 2012). Additionally, it has been shown that bivalent IgG binding to interdimeric band 3 potentiates the opsonizing IgG action on RBCs by recruiting C3b, a critical complement component, thus compensating for its low-affinity binding characteristic (Lutz, 2012).

The proportion of natural IgG antibodies bound to band 3 was found about a half of that associated to α-galactosyl residues, whereas the rest constituting about 70% of the total showed an unknown specificity (Sorette et al., 1991).

Another proposed hypothesis that originally had a great impact for involving a classical apoptosis hallmark, relates to the asymmetrical distribution of membrane phospholipids, a “sine qua non” characteristic of normal living cells. Of particular importance in this context is phosphatidylserine (PS), which accumulates in the inner membrane leaflet by action of an aminophospholipid translocase (APLT), a P_4_-type ATPase (Bitbol et al., 1987; Morrot et al., 1990). Externalization of PS is a widely accepted signal for macrophage recognition and phagocytosis of apoptotic and effete cells (Schroit et al., 1985; McEvoy et al., 1986; Fadok et al., 1992; Kiefer and Snyder, 2000; Segawa and Nagata, 2015). The specific recognition of surface exposed PS occurs by direct interactions with membrane macrophage receptors such as stabilin-2 (Park et al., 2008) or Tim-1/Tim-4 (Kobayashi et al., 2007) and indirect ones, via serum proteins that act as bridging opsonins such as lactadherin (Hanayama et al., 2002), thrombospondin-1, growth arrest-specific 6 (Gas-6) and protein S to α_*v*_β_3/5_ integrins and TAM tyrosine kinase receptors on macrophages (de Back et al., 2014; Segawa and Nagata, 2015).

In addition to the already mentioned exposure of natural antibodies-binding neoantigenic regions and surface PS, another unique signal for RBC removal has been proposed. This involves CD47, a transmembrane RBC glycoprotein that interacts with signal regulatory protein α (SIRP α) on macrophages blocking erythrophagocytosis (Oldenborg et al., 2000). However, in “artificially aged” RBCs (oxidatively treated) phagocytosis was stimulated by F(ab)_2_ anti-CD47, which prevented binding of oxidized CD47 to SIRPα. A similar effect seemed to be elicited by thrombospondin-1(TSP1), a known CD47 natural physiological ligand. It was therefore proposed that CD47 could act like a switch promoting or inhibiting phagocytosis, depending on whether TSP1 becomes bound or not (Oldenborg et al., 2000; Burger et al., 2012). It has been suggested that TSP1 binding to CD47 may be responsible for *in vivo* clearance of senescent RBCs.

An interesting hypothesis has been raised recently; drawing attention on the likely possibility that removal tagging signals on circulating RBCs may pass undetected because of their rapid dismissal. It was shown that the aging RBC decreases its membrane content of spectrin and flotillin-2, a lipid raft marker (Ciana et al., 2017). It was also found that vesicles induced by Ca^2+^-A23187 treatment were depleted of flotillin-2. It was proposed by above authors, that vesicles would contain a balanced lipid-bilayer/cytoskeletal protein ratio so that their release should occur without affecting the biconcave-disk shape of the cell. The hypothesis has been put forward that the continuous removal of vesicles by resident macrophages and the “pitting” splenic action during RBC aging, would reduce the cell size down to a minimum with a consequent increased rigidity (Ciana et al., 2017). This would lead to sequestration at the narrow splenic slits, recognition of accumulated tagging signals and finally clearance by phagocytosis.

On the other hand, earlier works stressed the importance of an elevated internal free Ca^2+^ as possible triggering signal for the events leading to clearance of senescent RBCs (Romero, 1978; Romero and Romero, 1999a; Bosman et al., 2005; Bogdanova et al., 2013). This idea finds support first, on the raised internal Ca^2+^ occurring during RBC aging as result of a steadily increased entry into cells having a progressive pumping deficiency (Romero and Romero, 1997, 1999b; Lew et al., 2007). Secondly, such a Ca^2+^ rise appears as common denominator of most of above mentioned age-related changes (Elgsaeter et al., 1976; Allan and Michell, 1977; Turrini et al., 1991; Kiefer and Snyder, 2000; Lang K. S. et al., 2003; Bogdanova et al., 2013).

Contrary to what would be expected from an abrupt clearance process, tagging signals steadily accumulate during the RBC lifespan. It is generally assumed that they trigger cell removal after reaching a threshold, as suggested for IgG binding where a few hundred molecules seem required (Bosman et al., 2005). In contrast with this view, previous work proposed a key role for the Gárdos channel (also known as KCNN4, KCa3.1, IKCa1) in the earlier events of senescent RBC clearance (Romero and Romero, 1999a). Accordingly, the channel would act as a molecular transducer between a monotonic signal (steadily rise in free internal Ca) and an “all-or-none” change (abrupt, self-generated Ca^2+^ increase, caused by membrane hyperpolarization due to channel opening) required for both a time-dependent sequestration and recognition of the aged cell. Essential to this view is the existent factual relationship between an increased Ca^2+^ content, activity of the Gárdos channel and cellular dehydration, referred to recently as the central paradigm of erythrocyte volume homeostasis (Martinac and Cox, 2017). Hence, a circulation-dependent raised cell Ca^2+^ above threshold levels (*caused by pressure or shear stress*) promotes erythrocyte dehydration by activating the Gárdos channel.

The purpose of the present study is to address the role that a raised free internal Ca^2+^ might possibly have on the clearance of aged cells, by studying *in vitro* its action on human erythrophagocytosis by peripheral monocyte-derived autologous macrophages under various experimental conditions. A complex action of Ca^2+^ was revealed, presumably involving phosphatidylserine externalization, IgG binding, complement activation and Gárdos channel activity.

## Materials and methods

All reagents of the best quality available were purchased mainly from Sigma-Aldrich Corp., St Louis, USA. Protein A-sepharose CL-4B was obtained from GE Healthcare, Uppsala, Sweden. Fetal calf serum (FCS) was from GIBCO BRL, New York, USA. The pH of all solutions was adjusted at room temperature (RT) to ± 0.02 units. Fresh human blood (mainly 0+) was used, obtained from healthy subjects of both sexes, equally represented and mostly with ages between 35 and 55 years old.

### Isolation of peripheral blood monocytes

Blood (10 ml) was collected by venipuncture in the presence of citrate (3.8%) and mixed with equal vol of phosphate-buffered saline (PBS) (310 mOsm/kg H_2_O, 150 mM NaCl + 20 mM (Na/K) phosphate buffer, pH 7.4), supplemented with 2 mM EDTA-Na_2_ and 10 mM glucose. The diluted blood was deposited on top of a ficoll-hypaque (FH) layer (density = 1.0775 g/ml) at a vol ratio blood/FH of 1:0.5 and spun down at 900 g for 30 min at RT. The cells banding at the serum-FH interphase were removed carefully and washed by resuspension in PBS medium supplemented as above and centrifuging at a sequentially decreasing low force (300, 200 and twice at 100 g). The fraction obtained consisted mainly of mononuclear cells (MNC) (>90%).

MNCs were resuspended in RPMI 1640-medium containing 0.03% glutamine and supplemented with 10% (vol/vol) heat-inactivated FCS plus antibiotics (100 U/ml penicillin G and 100 μg/ml streptomycin sulfate). Cells were enumerated in a Neubauer chamber and adjusted to a density of 3 × 10^6^ cells/ml with supplemented-RPMI medium (MNC suspension). Viable cells were routinely determined by trypan blue exclusion (Freshney, 1987). Viability always exceeded 98%.

To promote cell adherence and induce macrophage transformation, aliquots of the above MNC suspension (70 μl) were seeded on glass coverslips, deposited inside 6-holes culture dishes and placed in an air-CO_2_ incubator. After 1 h at 37°C under a 5% CO_2_-humidified air atmosphere, 2 ml of warm RPMI medium supplemented as above were added to each well and incubation further continued for up to 24 h.

### Loading erythrocytes with Ca^2+^

Erythrocytes from above FH fractionation (approx. 200 μl from the bottom of RBCs pellet) were separated and stored overnight in PBS plus 10 mM glucose and antibiotics. They were then washed once with PBS and thrice with a choline medium, containing (mM): choline Cl, 109; KCl, 5; Tris-HCl, 20 (pH 7.4). RBCs were loaded with Ca^2+^ by incubating in choline medium (1% hematocrit) for 30 min at 37°C under moderate shaking, in the presence of 10 μM A23187 for uniform Ca^2+^ permeabilization (Dagher and Lew, 1988) and 1 mM Ca-EGTA buffers, set at different Ca/EGTA ratios to obtain 0–20 μM ionized Ca (Fabiato and Fabiato, 1979). After loading, the ionophore was removed by washing once with 100 vols of choline medium containing 2 mg bovine serum albumin (BSA)/ml and twice with a similar medium but having 0.5 mg BSA/ml. RBCs were further washed twice with PBS to remove the remnant BSA and finally suspended in RPMI. Erythrocytes were enumerated in a Neubauer chamber and diluted with RPMI to about 50 times the number of monocytes/coverslip.

Since Mg^2+^ is also transported by A23187 (Reed and Lardy, 1972), this ion was omitted from the Ca^2+^-loading media in order to inactivate the Ca^2+^ pump. As a reference control, in some experiments Mg^2+^ was added to the loading medium at 0.15 mM, a concentration set at equilibrium with a −12 mV membrane potential (Flatman and Lew, 1980). Thereby, no net Mg^2+^ movements were expected during RBCs loading.

### Obtention of autologous serum (AS)

A parallel blood sample (10 ml) was collected without anticoagulant and allowed to clot at 37°C for 2 h. Thereafter, it was centrifuged at 3,000 g for 20 min at RT and kept at 4°C for 1 h before serum withdrawal. The AS was stored at −20°C until use on the following day.

### Affinity purification of IgG

Sepharose-coupled protein A was treated as recommended by the manufacturers and further used as described next. Briefly, it was washed several times with 50 mM Tris-HCl buffer (pH 7.0) by centrifuging 3 min at 500 g. One ml of this slurry sediment was packed at 15,000 g for 15 min. The supernatant was replaced by 500 μl of AS, the suspension mixed and left shaking for 15 min at RT. After packing as just described, the supernatant consisting of IgG-depleted serum was recovered, supplemented with antibiotics and preserved at −20°C until use. The total protein content of supernatants was usually 27–30 mg/ml.

The remaining sediment was washed five times with 50 mM Tris-HCl buffer (pH 7.0) and packed at 15,000 g as described before. To elute bound IgG, it was added 500 μl of 0.1 M citric acid (pH 3.0); the suspension mixed and left shaking for 15 min at RT. After which, the mixture was packed as above, the supernatant containing eluted IgG was recovered and neutralized to pH 7.0-7.5 by adding appropriate amounts of 1M Tris-HCl solution (pH 9.0). The IgG solution obtained was supplemented with (mM): NaCl, 150; CaCl_2_, 2; MgCl_2_, 1; plus antibiotics, and finally stored at −20°C until use. This solution generally contained 8–9 mg protein/ml. The efficiency of the process was checked by SDS-PAGE, using 10% polyacrylamide gels in the presence of 1% (vol/vol) 2-mercaptoethanol (Laemmli, 1970). Protein concentration was estimated using the Lowry method (Lowry et al., 1951). Equal amounts (20 μg) of protein were loaded per track for electrophoretic assays.

### Preparation of liposomes

Small unilamellar liposomes were prepared by sonication essentially as described by Fadok et al. (1992). The lipids, PS (L-α-phosphatidyl-L-serine) and L-α-dimyristoyl phosphatidylcholine (PC) were dissolved in chloroform at a molar ratio PS/PC of 3:7 and roto-evaporated at 50–60°C under reduced pressure. The dry material was resuspended in minimal medium of composition to be described later and vigorously shaken. The mixture was subsequently sonicated for two 10 min-cycles at 1°C, in a Braun-Sonic 2000 sonicator (Fisher Scientific Products, Pittsburgh PA). The clear liposomal suspension (2 mM total lipid) was stored at −20°C until use. Liposomes containing only PC were used as control.

### Erythrophagocytosis assays

After adherence, coverslips were washed five times with warm RPMI medium to remove non-adherent cells. The RBCs (loaded or not with Ca^2+^) were suspended in RPMI medium supplemented or not with 40% (vol/vol) AS or other additions. This suspension was deposited on macrophages-attached coverslips and incubated for 2 h at 37°C in a 5% CO_2_ humidified-air incubator. Thereafter, non-adherent RBCs were removed from coverslips by washing thrice with PBS and the attached cells were fixed with 2.5% glutaraldehyde in 0.15 M (Na/K) phosphate buffer (pH 7) for 15 min at RT. Subsequently, they were Wright-stained using conventional methods and observed under a light microscope (Nikon Eclipse E400).

In some experiments, macrophages were preincubated with PC- or mixed (PS + PC)-liposomes (200 μM total lipid content), for 30 min at 37°C in RPMI medium, in a 5% CO_2_ humidified-air incubator. After washing five times with warm RPMI medium, macrophages were challenged with 7 μM Ca^2+^-loaded RBCs and phagocytosis then assayed in a similar medium in the presence and absence of 40% AS, as described above.

Erythrophagocytosis was quantified (in percent) by scoring the number of macrophages having not only ingested but also attached RBCs, the latter considered an early step of the phagocytic process (Elliott and Ravichandran, 2010). At least 10^3^ macrophages counts were accumulated for each condition to minimize counting error, except for experiments with liposomes where 300 cells were scored.

In some assays, the RMPI medium employed for phagocytosis was replaced by a minimal medium containing (mM): NaCl, 140; KCl, 5; CaCl_2_, 2; MgCl_2_, 1; Hepes (pH 7.5), 10; glucose, 10; penicillin G, 100 U/ml and streptomycin, 100 μg/ml, leading to no significantly different results (not shown).

### Statistical analysis

Data were analyzed using GraphPad Prism Version 5.01 software for Windows. Statistical analysis was performed by unpaired two-tailed *t*-test when 2 groups were compared or 1-way ANOVA with Bonferroni post-tests when comparing more than 2 groups. Statistical significance of the data was defined as follows: *P* > 0.05 (n.s), *P* ≤ 0.05 (^*^), *P* ≤ 0.01 (^**^), *P* ≤ 0.001 (^***^). Each experiment presented corresponded to a different donor.

## Ethics

The present study was approved by the Bioethics Committee of the Faculty of Sciences, Central University of Venezuela. Investigations were carried out in accordance with the principles of the 2013 Declaration of Helsinki. Written informed consent was obtained from all blood donors participating in this study.

## Results

### Effects of Ca^2+^ and AS on erythrophagocytosis

Early work showed the requirement of both internal Ca^2+^ and presence of AS for phagocytosis of human RBCs by leukocytes *in vitro* (Romero and Romero, 1999a). This study, however, employed ghosts from the two cell types involved, a condition that may have obscured in part the conclusions drawn. With the interest of confirming these observations under more physiological conditions, intact RBCs were loaded with Ca^2+^ by means of the ionophore A23187 and exposed to monocyte-derived autologous macrophages. In addition, to assess a possible effect of Mg^2+^, phagocytosis was studied on cells treated or not with this ion during loading.

As reported in Table 1, with RBCs loaded in the virtual absence of both Ca^2+^ and Mg^2+^, the phagocytosis degree was about 2%, and tended to become reduced to a half when 0.15 mM Mg^2+^ was also added with the ionophore. Phagocytosis of these cells was significantly stimulated 2.5-fold by adding 40% AS to the assay medium whereas it remained unaltered if Mg^2+^ was present during loading.

**Table 1:** The effect on phagocytosis of Mg omission during erythrocyte loading.

**Additions to loading medium**			**Addition to assay medium**	***P***
**Ca^2+^ (μM)**	**0 Mg**	**0.15 Mg (mM)**	**AS (40%)**	
0	1.70 ± 0.85^a^ (6)	0.76 ± 0.23^e^ (3)	−	(a-b)^*^
				(a-e)^n.s^
				(a-g)^n.s^
	4.30 ± 2.65^b^ (6)	0.98 ± 0.23^f^ (3)	+	(e-f)^n.s^
				(b-f)^*^
				(b-d)^***^
20	14.6 ± 4.82^c^ (5)	–	−	(c-a)^***^
				(c-d)^*^
	27.0 ± 4.94^d^ (4)	–	+	(d-a)^***^
100	–	2.70 ± 0.30^g^ (3)	−	(g-h)^*^
				(g-e)^***^
	–	9.0 ± 2.54^h^ (3)	+	(h-f)^**^
				(h-b)^*^

*RBCs were loaded with and without Ca in choline medium, in the absence and presence of Mg. When Ca was present, it was added at 20 and 100 μM free concentrations in solutions lacking or not Mg, respectively. Loaded cells were then assayed for phagocytosis in RPMI medium to which autologous serum (AS) was added or not. The phagocytosis degree (in percent) is presented as mean value ± SD of mean from the number of experiments shown in parenthesis. The probability (P) derived from statistical comparison of the data indicated with letters is also given. The degree of significance rises with increasing number of asterisks and (n.s) denotes not significant. See the text for further details*.

In contrast, after loading with 20 μM Ca^2+^ and no Mg^2+^, phagocytosis increased high significantly by nearly 900% and became additionally stimulated 2-fold by adding serum (Table 1). On the other hand, with 100 μM Ca^2+^ and Mg^2+^ present during loading, phagocytosis was only enhanced by about 400% and serum almost trebled this effect. The extent of phagocytosis attained without AS in these cells was not significantly different from that of cells loaded in the nominal absence of divalent cations, thus suggesting they contain almost a comparable very-low ionized Ca concentration.

These results show first, that free internal Ca^2+^ is required for phagocytosis and that serum enhances its effect, thus confirming previous findings. Secondly, cells loaded in the nominal absence of both Ca^2+^ and Mg^2+^are more prone to become phagocytosed with serum present. This increased propensity to phagocytosis is also attained by omitting Mg^2+^ during Ca^2+^ loading. Thirdly, phagocytosis is markedly diminished when loading with high ionic Ca^2+^ in the presence of Mg^2+^.

Subsequent experiments were performed only on cells loaded with Ca^2+^ in the absence of Mg^2+^.

### Dependence of phagocytosis on intracellular Ca^2+^

With the interest of determining the dependence of phagocytosis on internal Ca^2+^, RBCs were loaded with 0, 0.5, 7, and 20 μM ionized Ca^2+^ concentrations. The process was then assayed in RPMI medium, to which 40% AS was added or not.

**Figure 1:**
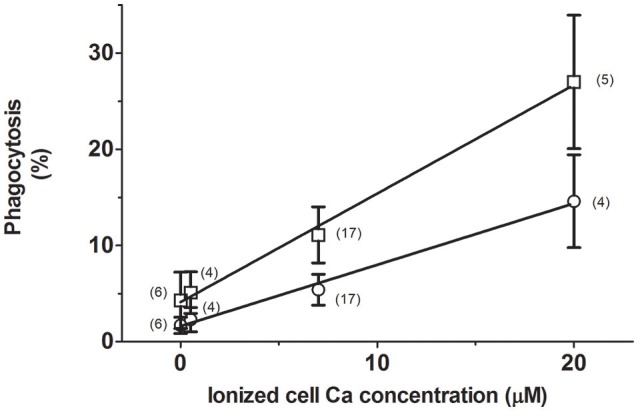
Stimulation of erythrophagocytosis by internal Ca and autologous serum. RBCs were loaded with 0, 0.5, 7, and 20 μM ionic Ca concentrations in a choline medium without Mg. The extent of phagocytosis (in percent) after incubation without (circles) or with 40% AS (squares) is given as mean values of the number of experiments shown within parenthesis. Vertical bars represent ± 1SD of mean. Collected results from different experiments are given. The curves drawn correspond to linear regression lines, whose determination coefficients (r^2^) are 0.997 and 0.994 for data from cells incubated with and without serum, respectively.

The extent of phagocytosis followed a strict linear relationship (correlation coefficient r^2^ = 0.994) with the free Ca^2+^ content of cells (Figure 1). Accordingly, it was increased from about 2 to nearly 15% by raising Ca^2+^ from 0 to 20 μM, respectively. Addition of serum further stimulated phagocytosis by almost 100%, while keeping the relationship linear (*r*^2^ = 0.997). These results clearly indicate that phagocytosis depends monotonically on the ionic cell Ca^2+^ content, whether in absence or presence of serum.

### Effects of preliminary incubation of macrophages with liposomes

The results presented above undoubtedly show that phagocytosis is stimulated by a rise in the free Ca^2+^ content of erythrocytes. This ion is a well-known modulator of PS externalization in human RBCs (Bitbol et al., 1987; Williamson et al., 1992), which in turn constitutes an important signal for macrophage recognition and erythrocyte removal (Schroit et al., 1985). To investigate if the Ca^2+^ action is related to PS exposure, macrophages were preincubated with PS-containing liposomes in RPMI medium. As control, macrophages were similarly exposed to PC liposomes. After washing, macrophages were challenged with 7 μM Ca^2+^-loaded RBCs, in the presence and absence of 40% AS.

As was expected, after exposing macrophages to PC-containing liposomes, the extent of phagocytosis was not much different to that obtained with untreated ones. Thus, the mean phagocytosis value from two separate experiments was 7.5 and 11.9%, without and with serum, respectively (compare with data of Figure 1). In marked contrast, after pretreating macrophages with PS-containing liposomes, the corresponding phagocytosis only amounted to 2.4 and 6.4%, respectively. These results demonstrate that phagocytosis of Ca^2+^-loaded RBCs is selectively affected by previous exposure of macrophages to a PS carrier, being almost fully blocked in the absence of AS whilst partially affected in its presence.

### Influence on phagocytosis of serum IgG removal and autologous IgG supply

To study whether stimulation of phagocytosis by AS depends essentially on the IgG content, the latter was selectively removed from serum by adsorption to protein A. This procedure appeared satisfactorily accomplished when monitored by SDS-PAGE analyses, producing two fractions: one corresponding to IgG-depleted serum and the other consisting of purified IgG. These were tested on RBCs loaded with 7 μM free Ca^2+^.

**Figure 2:**
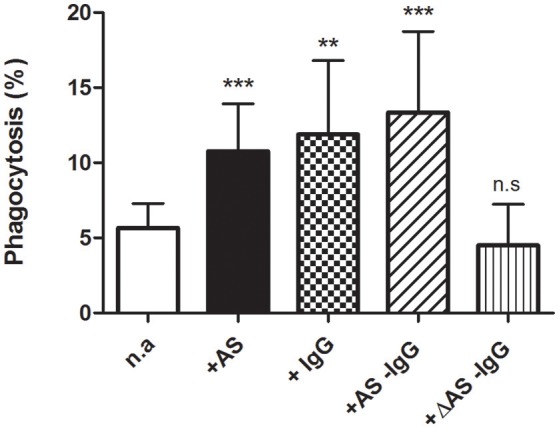
Parallel enhancement of erythrophagocytosis by IgG and serum IgG depletion. Phagocytosis of 7 μM Ca-loaded cells was assessed (in percent) after 2 h incubation in RPMI medium, with no addition (n.a) or after adding 40% autologous serum (+AS), 2 mg/ml purified IgG (+IgG), 40% IgG-depleted serum (+AS −IgG), or 40% IgG-depleted serum inactivated by heat (+ΔAS −IgG). Results from ten experiments are shown as mean value ± 1SD of mean, except for purified IgG and heat inactivation conditions where only four experiments are included. Vertical bars indicate the SD. Asterisks number denotes statistical significance and n.s stands for not significant. See the text for further details.

As was expected, phagocytosis increased high significantly from nearly 6 to about 12% after adding either 40% AS or 2 mg/ml purified IgG (Figure 2). To our surprise, it was similarly stimulated by IgG-depleted AS. This effect, however, was completely abolished by heating depleted serum for 30 min at 55°C. Thus, phagocytosis reached about 5% under this condition, a value not statistically different from that obtained without serum (Figure 2).

**Figure 3:**
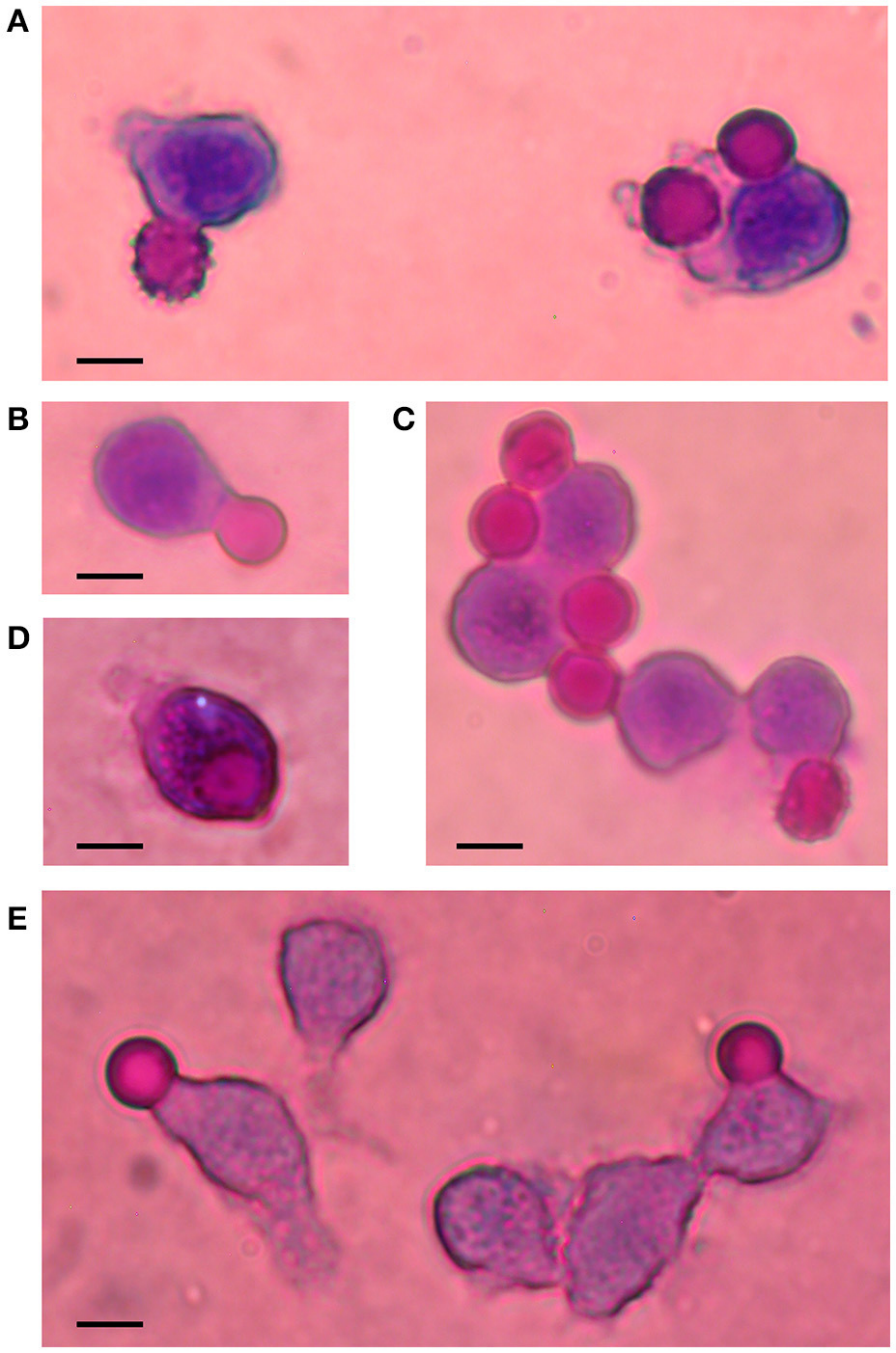
Phagocytosis of Ca^2+^-loaded RBCs. Illustrative images of Wright-stained cells are shown in this composite figure. Ca^2+^-loaded RBCs (7 μM) were incubated for 2 h in RPMI medium, in the absence **(A)** and presence of AS **(B)**, affinity-purified IgG **(C)**, IgG-depleted serum **(D)** and inactivated IgG-depleted serum **(E)**. Notice late stages of echinocytes: crenated and smooth spheres, undergoing phagocytosis. Bars represent 10 μm.

Microscopic observations showed that RBCs having different degrees of shrinking were phagocytosed following above treatments. Cell shapes varied from echinocytes to smooth spheres in spite that they were homogenously loaded with Ca^2+^. No preferential shape to be phagocytosed was evident. Figure 3 illustrates some aspects of this process under the various conditions studied.

The above results demonstrate that the stimulatory action of AS can be replaced with equal potency by affinity-purified autologous IgG. The findings also show that enhancement of Ca^2+^-dependent phagocytosis by IgG-depleted serum seems related to complement activity.

### Action of Gárdos channel blockers

Early work has proposed an involvement of the Gárdos channel in the physiological dismissal of senescent RBCs (Romero and Romero, 1999a). Therefore, it was of interest to assess the effect on phagocytosis of some inhibitors of this channel. When incorporated, they were present throughout the whole experimental procedure, at concentrations at least 10 times higher than their corresponding IC_50_. Two selective blockers were chosen. The potent inhibitor clotrimazole (CLT) was the first to be tested on RBCs loaded with 0.5 and 7 μM ionized Ca. Phagocytosis was assayed with and without 40% AS, in the presence and absence of 2 μM CLT.

**Figure 4:**
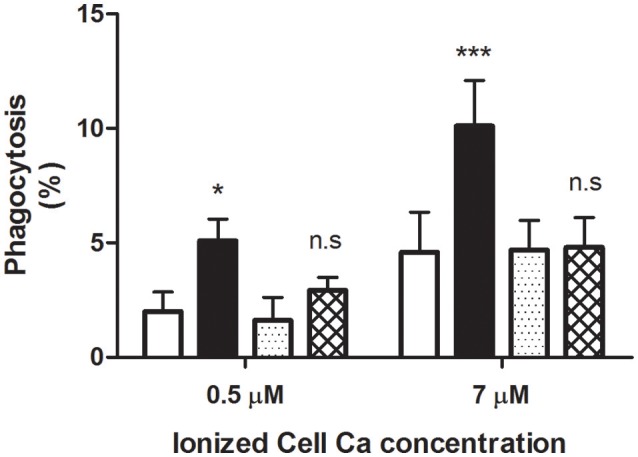
Blocking by clotrimazole of serum-dependent erythrophagocytosis. RBCs were loaded with 0.5 and 7 μM ionic Ca concentrations in a choline medium, with and without 2 μM clotrimazole. Phagocytosis of loaded cells was determined (in percent) following incubation in RPMI medium without (white and dotted columns) and with 40% AS (black and cross-hatched columns), in the presence (dotted and cross-hatched columns) and absence (white and black columns) of clotrimazole. Cells were treated with the azole throughout the whole experiment. Results from five experiments are shown as mean value ± 1SD of mean. Vertical bars indicate the SD. Asterisks number denotes statistical significance and n.s stands for not significant. See the text for further details.

As expected, adding AS to 7 μM Ca^2+^-loaded cells brought about a highly significant stimulation of phagocytosis to nearly 10% (Figure 4). Remarkably, this action was fully blocked by CLT. Thus, the extent of phagocytosis obtained with serum plus CLT was about 4%, a value not statistically different from that of control cells without serum. These results were reproduced employing RBCs loaded with 0.5 μM Ca^2+^, but a lower phagocytosis degree was attained (Figure 4).

Almost identical findings were obtained by replacing CLT with TRAM-34, a highly selective channel blocker. Accordingly, in three separate experiments on cells loaded with 7 μM Ca^2+^, phagocytosis (in percent ± 1 SD of mean) was significantly increased from 5.6 ± 1.35 to 10.1 ± 2.08 (*P* < 0.05) when 40% AS was added. In contrast, in the presence of 100 nM TRAM, the extent of phagocytosis without and with serum did not differ statistically from each other (*P* > 0.05), amounting to 4.4 ± 1.53 and 2.7 ± 2.15, respectively.

The above results clearly demonstrate that Gárdos channel blockers inhibit selectively the stimulatory activity of AS on phagocytosis, strongly indicating an involvement of this channel.

## Discussion

A common finding with normal human RBCs is the increase of internal Ca^2+^ that occurs during cell aging (Shiga et al., 1985; Aiken et al., 1992; Romero et al., 1997; Lew et al., 2007), thus suggesting a cause-effect interrelation. Modification of internal Ca^2+^ by ionophore loading was employed here as a model for studying some aspects of RBC aging leading to phagocytic clearance. Accordingly, cells were loaded by incubating with EGTA-buffered low-μM free Ca^2+^ concentrations instead of mM levels (i.e., 1–2 mM), that most likely would promote eryptosis (Lang K. S. et al., 2003; Lang et al., 2005; Romero, 2011). These cells were subsequently challenged with activated macrophages under various conditions, to gain insight into the mechanisms through which Ca^2+^ might promote erythrophagocytosis.

### Effect on phagocytosis of Mg^2+^ omission and influence of Ca^2+^

In order to establish a reliable relation between phagocytosis and free internal Ca^2+^, loading with this ion was accomplished by using the divalent cation selective ionophore A23187 in Mg^2+^-free choline medium, which would simultaneously deplete RBCs of Mg^2+^. It should be stressed that by lacking ATP-Mg, the true Ca^2+^ pump substrate, Mg^2+^-depleted cells are incapable of extruding Ca^2+^ (Schatzmann and Vincenzi, 1969). Therefore, internal Ca^2+^ is kept constant during phagocytosis. We did not attempt to measure intracellular Ca^2+^ in these cells. However, the ionized concentration should equal that present in the loading medium, if no major changes of internal pH take place.

On the other hand, the lack of Mg^2+^ during loading with A23187 in Na-containing media causes RBC membrane disturbances leading to increased mechanical fragility and enhanced permeability (Romero, 1974). The final outcome is hemolysis since the ionophore also catalyzes rapid Na transport in the complete absence of divalent cations (Flatman and Lew, 1977). These effects are markedly reduced or blunted by replacing Na by choline or adding low Mg^2+^ or Ca^2+^ concentrations to the loading medium (Flatman and Lew, 1977).

The present study showed that RBCs exposed to ionophore in the virtual absence of both Ca^2+^ and Mg^2+^, were phagocytosed to an equal low-extent than cells whose free internal Mg^2+^ was kept at normal levels by adding Mg^2+^ during loading, thus suggesting a comparable cell behavior. However, such a basal phagocytosis was increased significantly on AS addition whilst it was not affected if Mg^2+^ was present during loading. This observation indicates that some membrane alterations may have taken place in (Ca^2+^ + Mg^2+^)-depleted cells, despite they apparently maintained normal integrity and permeability after ionophore removal. Since these changes appeared evident on serum addition, it seems likely that new antigenic epitopes or binding sites became accessible on these cells.

Confirming and extending previous findings (Romero and Romero, 1999a), basal phagocytosis was stimulated significantly by rising ionized Ca in Mg^2+^-depleted RBCs. Of note, erythrocytes loaded with 100 μM Ca^2+^ in the presence of Mg^2+^ were less prone to phagocytosis than those cells loaded with five times less free Ca^2+^ but in the absence of Mg^2+^. Such dissimilarities in phagocytic extents can be attributed indeed to active Ca^2+^ extrusion. In spite of a rapid ATP breakdown occurring by Ca^2+^ pump activity during loading, Mg^2+^-containing cells would restore their ATP content through glycolysis as substrates become available during cell handling and phagocytosis. Under such conditions, however, the amount of free Ca^2+^ remaining in cells is unknown.

The extent of phagocytosis attained after loading Mg^2+^-depleted cells with 0.5 μM free Ca^2+^, was not much at variance with that reported for young human RBCs employing a roughly comparable phagocytosis model (Luján-Brajovich et al., 2009). This may lend some support for validating the use of above cells in our present study.

The results shown here disclose Ca^2+^ as first messenger of a chain of events leading to phagocytosis. Accordingly, its action appears multiple, promoting activation of various processes such as PS externalization, IgG binding, complement deposition and Gárdos channel activity, which shall be discussed separately.

### Dependence of phagocytosis on Ca^2+^-associated PS exposure

Our results have clearly shown that phagocytosis of Mg^2+^-depleted, Ca^2+^-loaded cells was blocked after preincubating macrophages with liposomes that contained a mixture of PS plus PC. No such an effect was found when they were made of PC as the only phospholipid. Remarkably, phagocytosis was almost fully inhibited in the absence of AS whilst partially affected in its presence. These results suggest that two distinct processes are involved: one that occurs with no serum present and which is selectively blocked by PS. The other seems unrelated to PS and expressed in presence of serum, as referred to later. These findings are in agreement with the stereospecific inhibition of apoptotic-lymphocyte phagocytosis by liposomes containing the L-serine form of PS (Fadok et al., 1992). They strongly indicate that PS externalization mediates phagocytosis of Mg^2+^-depleted cells loaded with Ca^2+^.

Since APLT is inactive in above cells due to both Ca^2+^ presence and lack of ATP-Mg, the true enzyme substrate (Morrot et al., 1990), stimulation by Ca^2+^ of phospholipid scramblase (TMEM16F) readily leads to PS exposure (Williamson et al., 1992; Bratton et al., 1997; Williamson, 2015). This appears the main mechanism for PS externalization on Mg^2+^-depleted RBCs. Contribution of other known mechanisms seems unlikely for the following considerations. First, it is recognized that Gárdos channel activity promotes PS exposure on human RBCs (Lang P. A. et al., 2003; Wesseling et al., 2016). However, a similar action on Ca^2+^-loaded, Mg^2+^-depleted cells is highly improbable since phagocytosis was unaltered by adding Gárdos channel inhibitors in the absence of AS, as shall be discussed later. Additionally, such findings also discard the possibility of a shrinkage-related PS externalization, as reported for RBCs under hyperosmotic shock (Lang et al., 2004). Secondly, it is known that protein kinase Cα activity is involved in PS exposure on human RBCs (de Jong et al., 2002). Nonetheless, recent work showed that selective enzyme inhibitors (chelerytine, calphostin) hardly affect the PS externalization induced by A23187 plus Ca^2+^ in above cells (Wesseling et al., 2016), thereby indicating that protein kinase Cα is not responsible for PS scrambling in Ca^2+^-loaded, Mg^2+^-depleted RBCs.

Significantly, due to the intrinsic characteristic of being Mg^2+^-depleted cells, their phagocytosis dependence on PS exposure disclosed by our aging model, roughly resembles that of eryptosis, a process evoked at much higher Ca^2+^ levels (Lang et al., 2005). Unlike with the latter, however, the extent of PS externalization does not increase during the normal RBC lifespan (Boas et al., 1998; Lutz, 2004; Willekens et al., 2008; Ghashghaeinia et al., 2012; Franco et al., 2013), thus demonstrating that it is not tagging signal for clearance of senescent RBCs. Nonetheless, it is still an open question as to whether PS becomes exposed on those cells already sequestered, that become inaccessible to analyses. Such an answer obviously cannot be tested experimentally for ethical reasons. Perhaps, the cell model used here may help in approaching this problem.

On the other hand, erythrocyte vesicles bearing PS and IgG are shed from the membrane during the normal RBC lifespan, in a way presumably associated to PS externalization (Willekens et al., 2008). Microvesicles are also produced when RBCs are exposed to the combined action of A23187 plus Ca^2+^ (Allan and Michell, 1977). We have observed no vesicles in our preparation that may have interfered with the phagocytosis assay as PS-containing liposomes did. Most probably, this was due to their dismissal by the low centrifugal force employed for cell wash following ionophore loading.

### Action on phagocytosis of serum IgG removal and autologous IgG supply

Very early work has shown the need of AS for promoting erythrophagocytosis by leukocytes *in vitro* (Greendyke et al., 1963). It is also widely known the general requirement of erythrocyte opsonization for phagocytosis by professional macrophages (de Back et al., 2014). In accordance with this assertion and confirming previous work (Romero and Romero, 1999a), phagocytosis of Ca^2+^-loaded RBCs was further enhanced by addition of 40% AS. This effect, like that attained without serum, exhibited a linear relationship with increasing ionized Ca, thus revealing a monotonic nature of activation. At all Ca^2+^ concentrations tested the magnitude of stimulation by AS doubled that obtained in its absence. We did not explore other serum concentrations, not knowing if that presently employed was the optimal.

Notably, the extent of phagocytosis reached with either 2 mg/ml purified IgG or 40% IgG-depleted serum, was practically identical to that attained with AS. The former IgG concentration is roughly equivalent to that of IgG present in 40% AS (about 3 mg/ml). These findings demonstrate on the one hand, that serum can be replaced by IgG stimulating phagocytosis with equal potency, thereby suggesting that its action can be accounted for by its IgG content. On the other hand, they also show that phagocytosis can be equally enhanced with similar potency by serum in a non-IgG dependent way. The latter action was suppressed by preheating IgG-depleted serum under conditions well established to inactivate the complement system (Soltis et al., 1979); strongly suggesting that complement activity is involved in such stimulation. In addition, the findings indicate that IgG is not needed for complement-stimulated phagocytosis of Mg^2+^-depleted Ca^2+^-loaded RBCs. This action was not studied further. Other normal serum factors that may be required for erythrophagocytosis could also be affected by heating.

Taking into consideration all preceding findings it becomes evident that at least, Ca^2+^ promotes phagocytosis of Mg^2+^-depleted RBCs via three apparently independent processes. The first is elicited in the absence of serum, and is mediated through PS exposure. The second is related to AS stimulation and presumably is mediated mostly by IgG, and the third one, corresponds to that associated with complement activation. The latter two processes seem to exert a sort of additive action on the former.

### Erythrophagocytosis inhibition by Gárdos channel blockers

The main finding of the present work was the action on phagocytosis of two Gárdos channel inhibitors. First, the azole containing compound CLT that at 2 μM, completely inhibited serum-dependent phagocytosis of cells loaded with 0.5 and 7 μM free Ca^2+^. This compound is a well-known potent channel inhibitor (IC_50_ = 50 nM in normal RBCs) (Alvarez et al., 1992). Though selective, however, it is a non-specific inhibitor since its interactions with a wide number of unrelated targets possessing dissimilar CLT affinities have been reported (Thomas et al., 1999; Klokouzas et al., 2001; Zhang et al., 2002).

The second blocker used, TRAM-34, is a triphenylmethane compound possessing a pyrazole moiety instead of an azole one, for which neither inhibits cytochrome P450-dependent enzymes nor exhibit the toxic CLT side effects. TRAM-34 is a highly-selective Gárdos channel inhibitor, having an IC_50_ = 20 nM (Wulff et al., 2000). This compound at 100 nM fully inhibited the AS-dependent phagocytosis of 7 μM Ca^2+^-loaded RBCs, thus confirming above findings with CLT.

The inhibitory effect of CLT and TRAM-34 just described clearly demonstrates a specific participation of the Gárdos channel in erythrophagocytosis. As these compounds fully blocked phagocytosis only in the presence of AS, the results disclose a peculiar involvement of this channel.

### Possible involvement of the Gárdos channel in erythrophagocytosis

It is widely recognized that band 3 aggregations occurs in RBCs exposed to oxidative stress, with resultant IgG binding and complement deposition (Low et al., 1985; Lutz et al., 1987; Turrini et al., 1991; Lutz, 2012). Based on these findings, some consensus has been reached for the proposal that oxidative stress, acting via band 3 peroxidation of cytosolic domain and concomitant binding of met-hemoglobin and hemichromes, may be the physiological trigger for such aggregation (Low et al., 1985; Arese et al., 2005; Lutz, 2012; Lutz and Bogdanova, 2013; Mohanty et al., 2014). In addition to these effects, oxidative stress or defects of antioxidative defense also enhance RBC Ca^2+^ entry (Lang K. S. et al., 2003), thus involving this ion in the above process.

It is quite feasible that Ca^2+^ can promote band 3 aggregation in Mg^2+^-depleted cells, as demonstrated by its action on the distribution of intramembrane particles in human RBC ghosts (Elgsaeter et al., 1976), believed to consist of band 3 macromolecular complexes (Verkleij and Ververgaert, 1978). Interaction of Ca^2+^ with cytoskeletal proteins is known to loosen the cytoskeleton network, weakening its anchorage to integral membrane proteins (Bogdanova et al., 2013). Consequently, a larger number of band 3 dimers are freed to move within the lipid bilayer plane, becoming capable of forming multimers and higher aggregates.

It is conceivable that upon Gárdos channel activation, the consequent membrane deformation and presumably hydrophobic mismatch imposed by dehydration, acting in concert with the loosening of band 3 cytoskeletal anchorage, may drive clustering of band 3 aggregates. The latter would lead to an increased IgG binding and presumably complement deposition, thus signaling macrophages for recognition and phagocytosis (Turrini et al., 1991; Lutz, 2004; Arese et al., 2005). This may explain the selective inhibition of AS-dependent phagocytosis by Gárdos channel inhibitors reported in the present study, and place the findings into a physiological context.

It is quite remarkable that a wide variety of hemolytic anemia, including sickle cell disease, thalassemia, Gárdos channelopathy, and both hereditary spherocytosis and xerocytosis, are associated to RBC Ca^2+^ overloading (Bookchin et al., 1988; Lew et al., 2002; Fermo et al., 2017; Hertz et al., 2017). The fate of such pathological cells, like that of senescent cells is to become phagocytosed by macrophages. Thus, it is not surprising a convergence of tagging signals in these cells for macrophage clearance. Aging and eryptosis may share the same final mechanisms for RBC dismissal (Romero, 2011). Along the same idea, recent work have put forward the hypothesis that an increased internal Ca^2+^ is the common component in the mechanism causing an accelerated RBCs clearance in some hemolytic anemia (Hertz et al., 2017).

In conclusion, the results presented in this work indicate that a rise in free internal Ca^2+^ is fundamental for promoting phagocytosis by autologous macrophages *in vitro*.

## Author contributions

PR and CH-C contribute equally in designing, performing and analyzing the experimental data.

### Conflict of interest statement

The authors declare that the research was conducted in the absence of any commercial or financial relationships that could be construed as a potential conflict of interest.
